# Performance of machine learning versus the national early warning score for predicting patient deterioration risk: a single-site study of emergency admissions

**DOI:** 10.1136/bmjhci-2024-101088

**Published:** 2024-12-04

**Authors:** Matthew Watson, Stelios Boulitsakis Logothetis, Darren Green, Mark Holland, Pinkie Chambers, Noura Al Moubayed

**Affiliations:** 1 Department of Computer Science, Durham University, Durham, UK; 2 Department of Public Health and Primary Care, Cambridge University, Cambridge, UK; 3 Division of Cardiovascular Sciences, The University of Manchester, Manchester, UK; 4 Department of Renal Medicine, Northern Care Alliance NHS Foundation Trust, Salford, UK; 5 School of Clinical and Biomedical Sciences, University of Bolton, Bolton, UK; 6 School of Pharmacy, University College London, London, UK; 7 Evergreen Life Ltd, Manchester, UK

**Keywords:** Medical Informatics, Information Technology, Outcome Assessment, Health Care

## Abstract

**Objectives:**

Increasing operational pressures on emergency departments (ED) make it imperative to quickly and accurately identify patients requiring urgent clinical intervention. The widespread adoption of electronic health records (EHR) makes rich feature patient data sets more readily available. These large data stores lend themselves to use in modern machine learning (ML) models. This paper investigates the use of transformer-based models to identify critical deterioration in unplanned ED admissions, using free-text fields, such as triage notes, and tabular data, including early warning scores (EWS).

**Design:**

A retrospective ML study.

**Setting:**

A large ED in a UK university teaching hospital.

**Methods:**

We extracted rich feature sets of routine clinical data from the EHR and systematically measured the performance of tree- and transformer-based models for predicting patient mortality or admission to critical care within 24 hours of presentation to ED. We compared our proposed models to the National EWS (NEWS).

**Results:**

Models were trained on 174 393 admission records. We found that models including free-text triage notes outperform structured tabular data models, achieving an average precision of 0.92, compared with 0.75 for tree-based models and 0.12 for NEWS.

**Conclusions:**

Our findings suggests that machine learning models using free-text data have the potential to improve clinical decision-making in the ED; our techniques significantly reduce alert rate while detecting most high-risk patients missed by NEWS.

**WHAT IS ALREADY KNOWN ON THIS TOPIC**

**WHAT THIS STUDY ADDS**

- Our study shows that, when used with transformer-based machine learning techniques, the rich patient data collected in EHR (including free-text triage notes) can significantly outperform the National Early Warning Score when predicting patient deterioration.

HOW THIS STUDY MIGHT AFFECT RESEARCH, PRACTICE OR POLICY

- Our work highlights the efficacy of machine learning for clinical decision support tools and the currently untapped information contained in free-text triage note data. Free-text data could be used in other areas of research to investigate whether similar improvements are possible.

## Introduction

 Early recognition and intervention of deteriorating patients is vital to prevent avoidable hospital deaths.^1^ Track and trigger systems, such as early warning scores (EWS), were developed to meet this need, providing a single aggregated score from a patient’s vital signs. Score thresholds define recommended response levels and urgency. EWS are used throughout a patient’s hospital admission pathway, from initial evaluation in the ambulance to emergency department (ED) triage, and subsequent monitoring on a ward.^2^ The UK’s Royal College of Physicians recommends the National Early Warning Score 2 (NEWS2),^3^ with aggregated scores of 0–20 indicating risk of death, cardiac arrest or critical care admission. Higher scores carry recommendations for appropriate clinical responses, for example, scores ≥7 mandate urgent senior clinical review.

EWS, calculated from vital signs, are part of the comprehensive assessment for patients presented to an ED.^3^ While NEWS alone is not recommended as a triage tool, it is included in patient streaming guidance, for example, in identifying patients suitable for same day emergency care.^4^ Since the introduction of manual early warning scores, such as NEWS, hospitals have reported a 20% reduction in mortality from sepsis and acute illness and a 50% reduction in in-hospital cardiac arrests.^5^ However, manual EWS only consider only a small number of parameters, have been shown to underperform in some patient groups and have low sensitivity (sensitivity NEWS: 0.12).^6 7^

Electronic health records (EHR) enable real-time detailed patient-level data collection, supporting machine learning development for patient care in areas such as admission, deterioration and mortality prediction.^8^ Machine learning systems can analyse much more data compared with simple decision tools, such as NEWS. Examples range from shallow models, such as gradient boosted decision trees (GBDT) for predicting mortality,^9^ to phenotyping,^10^ risk stratification^8^ and simple natural language analysis of presenting complaints to predict mortality or cardiac arrest.^11^

Unfortunately, the data collected, and its quality, varies significantly between EHR systems,^12^ hindering predictive model generalisability.^13^ The rigid data requirements of many machine learning techniques are incompatible with the real-world challenges of non-standardised data collection.^14^ Additionally, concerns around the generalisability, trustworthiness, safety and fairness must also be addressed before deployment in clinical settings.^13 15^ Most research focuses on structured tabular data,^16^ that is, numerical and categorical data, neglecting routinely recorded unstructured text data such as emergency triage notes. ED triage notes, often the earliest text data captured for non-elective admissions, vary greatly in content, from brief descriptions to extensive accounts including symptoms, social context, vital signs and physical examination findings. The ubiquity of text data makes it a natural contender for inclusion in deep learning models.

We investigated using modern natural language processing to incorporate unstructured free-text triage notes into deep learning models for predicting imminent clinical deterioration in emergency admissions. Previous studies used structured data only for this task.^16^ We systematically compare the performance of various feature sets (structured only, unstructured only, combined) and modelling techniques, including GBDTs and transformers.

Unstructured data hold significant promise for improved performance and generalisability across settings with differing data formats and treatment patterns.^17^ This study aims to discover useful information in this untapped resource and suggest future research avenues. Our experiments are designed for real-world clinical applications, evaluating potential clinical utility through the lenses of explainability, bias, fairness, and privacy, to create a viable decision-support tool fitting clinical workflows. This study:

- Develops and validates machine learning models for predicting the risk patient deterioration on a dataset consisting of 174 393 admission records and is the first study to explore the use of ED triage notes in machine learning models for patient risk stratification, moving beyond the traditional reliance on structured data.
- Systematically compares the utility of increasingly rich feature sets for patient deterioration risk modelling to identify the most useful features and possible points of implementation in clinical pathways.
- Asks whether our proposed techniques outperform the baseline risk assessments of NEWS and existing machine learning–based techniques.
- Investigates the use of explainability techniques to understand the impact different features have on the model and discusses the effect this has on model transparency.
- Investigates the bias of our techniques and compares this with NEWS.

## Methods

### Setting

Salford Royal Hospital has over 100 000 ED attendances and approximately 40 000 unplanned admissions annually. The hospital has used an EHR system, Allscripts, since September 2013 that captures patient data in real time from arrival at the ED, until discharge from hospital.

### Study design

A retrospective observational cohort study of routinely collected patient data from a single UK university teaching hospital. Three machine learning models were trained to predict critical deterioration within 24 hours of admittance to the ED: Light Gradient Boosted Machines (LightGBM),^18^ Bidirectional Encoder Representations from Transformers (BERT)^19^ and BioClinicalBERT.^20^ TRIPOD+AI guidelines^21^ were followed for reporting.

### Data collection and preparation

We extracted retrospective data from the EHR of all patients presenting to the ED between 1 April 2014 and 30 December 2022. We restricted the dataset to patients aged ≥18 years with a documented NEWS who were either admitted to the acute medical unit or received ambulatory emergency care or same-day emergency care.^4^ Planned admissions and day cases were excluded, as well as patients that received ward-based critical care interventions, such as invasive ventilation or cardiopulmonary resuscitation. Online supplemental table 1 describes all features collected by the system; not all are suitable for use in an early warning system, for example, ward utilisation is unknown at the time of presentation. Online supplemental table 2 describes valid ranges for manually recorded features. Online supplemental section 1.1 and table 1 detail the subsets of features used in the modelling.

**Table 1:** Feature sets used throughout experimentation

Feature set	Tabular features	Text features
Core tabular	Patient demographics; vital signs at admission Subset of bloods at admission: haemoglobin, urea, sodium, potassium, creatinine; main diagnosis, readmission, admit method and admission specialty	None
Extended tabular	Core tabular+admission blood tests Waterlow score^33^ CFS score^34^ Charlson index	None
Triage notes	Patient demographics	Triage note, presenting complaint
Text embeddings	Patient demographics, triage note embeddings, presenting complaint embeddings	None

All text embeddings are computed from a pretrained BioClinicalBERT^16^ model. See Data Collection and Preparation for full descriptions of vitals and blood tests included.

CFS, Clinical Frailty Scale.

As our aim was to create a system to support triage in the ED, we only used admission data. Blood tests were only taken when clinically indicated. Comorbidity and previous admission data were available for patients with prior admissions. Unstructured free-text data entered by the triage clinician was used.

To supplement recorded features, we constructed new features using recorded values, aiming to enhance the clinical information available; for example, the conversion of raw International Classification of Diseases, Tenth Revision, three number codes to their English name, as the full-text description includes more information that can be used by language models. Online supplemental section 1.2 details all engineered/augmented features.

#### Data labelling

Our tracked outcome was a composite of in-hospital mortality and/or critical care admission within 24 hours of presentation to the ED, aligning with previous studies for direct comparisons.^16^ This outcome also directly aligns with the development of EWS, including NEWS2.^3 22^ Specifically, our models predict patients at risk of experiencing a critical event defined as admissions where: the discharge or end-of-episode record indicates the patient died in the hospital *and* the record’s timestamp is within 24 hours of the admission timestamp *or* their service utilisation indicates admission to critical care or provision of critical interventions on the ward *and* this occurred within 24 hours of the admission timestamp.

#### Missing data and data imputation

While previous studies have analysed the effect of imputation of missing data,^16^ we focused on two machine learning modelling techniques (Machine Learning Models Section) that can handle missing data without imputation.

### Machine learning models

We compared GBDT, which provide state-of-the-art results on tabular data,^16^ with transformers, which represent the current state-of-the-art in text-based modelling.^19^ Despite fundamental architectural differences, both can be embedded in largely the same modelling workflow. Online supplemental figure 1 is our transformer-based modelling pipeline. We modify an earlier modelling pipeline^16^ to accommodate the novel features included in our dataset, as described in (online supplemental file 1, online supplemental section 1.3).

We focused our tree-based experiments on LightGBM decision trees,^18^ as they set the state-of-the-art on tabular data, often outperforming neural networks.^10 16^ Details on LightGBM training/validation are in online supplemental section 1.3.1.

We also experiment with two models using free-text data: BERT,^19^ trained on a general text corpus, and BioClinicalBERT,^20^ further trained on Medical Information Mart for Intensive Care clinical notes.^23^ Online supplemental section 1.3.2 outlines the training of transformer-based models.

#### Model evaluation

We partitioned samples chronologically 2:1 into training/validation sets. Given the nature of the ED, some patients in the validation set may have prior admissions in the training set due to being repeat attendees.^24^ As such, we also evaluated on a version of the validation set with all repeat patients removed. Results reports set sizes and demographics.

Our task was significantly imbalanced, with only 5% of patients experiencing a critical event. Thus, we preferred evaluation metrics that were robust to class imbalances and had previously been used for healthcare machine learning models.^16^ Our main metric was average precision (AP), calculated as the area under precision-recall (PR) curve, which is better suited to imbalanced tasks than the receiver operating characteristic (ROC) curve,^16^ although we include the latter for comparison. We also report the specificity of the model.

We avoided prescribing a specific decision threshold, as this requires additional clinical, operational and ethical considerations^25^; our chosen metrics measure discriminative skill agnostically of thresholds. However, we report F2 scores under a threshold of 0.5 to demonstrate possible model performance. To assess the clinical benefit of the model, we plot and analyse decision curves.^25^ All metrics are fully explained in online supplemental section 1.4.

#### Model explainability

To address the lack of explainability of our chosen architectures, particularly transformers, we used SHapley Additive exPlanations (SHAP) to calculate feature importance. SHAP enables in-depth analysis of model behaviour and can uncover hidden bias and spurious correlations (see Section 3). Techniques used to compute SHAP are explained in online supplemental section 1.4.1.

#### Model bias

To evaluate our models’ ability to produce fair outcomes across patient subgroups, we examined any unintentional bias introduced during training. We assessed group-based fairness, that is, performance differences between demographic groups, and individual fairness, that is, treatment of patients with similar expected outcomes, using generalised entropy index^26^ *I*, which encompasses both notions of fairness. Formulae for computing *I* are outlined in online supplemental section 1.4.2.

### Ethics

Local ethical approval to use the data was provided by Salford Royal Hospital’s Research and Innovation Department (21HIP13). Only non-identifiable, anonymised patient-level data collected in routine clinical practice are used, as its use does not breach confidentiality. Data were pseudonymised prior to release.

### Patient and public involvement

As this was an initial study into using machine learning and free-text features to augment NEWS, no patient and public involvement was conducted.

## Results

Of 381 687 extracted records, 81 367 booked admissions, elective admissions, maternity and elective trauma cases were removed. 125 926 non-acute medical admissions were also removed, leaving 174 393 emergency admissions comprising 86 215 unique patients. Removing repeat patients in the unseen validation set excluded 11 237 patients. Online supplemental figure 2 shows age and sex distributions. 90% of records are White British, 4.3% other White background and 5.7% are from other ethnic backgrounds. There was a high rate of missing data; patients with a missing NEWS score had lower mortality, were younger and had shorter stays.

Table 2 reports all performance metrics, and online supplemental table 3 reports performance metrics on the validation set with repeat patients removed, demonstrating similar performance. Table 2 and online supplemental table 3 also compare performance metrics for NEWS on the same sets of records.

**Table 2:** Full table of results for all LightGBM, BERT and BioClinicalBERT models tested on the entire validation set

Model architecture	Features	Precision	Recall	AUROC	F2	Specificity	AP
LightGBM	Core tabular	0.6755	0.1312	0.9043	0.1554	0.8188	0.3933
	Extended tabular	0.8728	0.4279	0.9560	0.4764	0.9174	0.6995
	Core tabular+text embeddings	0.8743	0.2829	0.9133	0.3271	0.9983	0.5272
	Extended tabular+text embeddings	0.9273	0.4140	0.9619	0.4656	0.9975	0.7482
	Text embeddings	0.8287	0.0430	0.7667	0.0531	0.9996	0.2465
BioClinicalBERT	Core tabular	0.5925	0.8425	0.9309	0.7769	0.9754	0.8003
	Extended tabular	0.4146	0.7426	0.9398	0.6412	0.9494	0.6569
	Triage notes+demographics	0.1288	0.9136	0.9056	0.4189	0.7014	0.5545
	Core tabular+triage notes	0.7539	0.9222	0.9791	0.8828	0.9814	0.9188
	Extended tabular+triage notes	0.8741	0.9202	0.9665	0.9106	0.9936	0.9244
BERT	Core tabular	0.2879	0.8962	0.8583	0.6300	0.8757	0.2548
	Extended tabular	0.473	0.3446	0.8043	0.3664	0.9814	0.3090
	Triage notes+demographics	0.2807	0.4756	0.7571	0.4176	0.9316	0.2226
	Core tabular+triage notes	0.3742	0.9741	0.8060	0.2845	0.3191	0.4143
	Extended tabular+triage notes	0.3891	0.9759	0.8241	0.3264	0.4408	0.4386
NEWS	NEWS scores	0.2816	0.1206	0.6617	0.1361	0.9797	0.1239

For each metric, green highlights the best performing model, and red indicates models that perform worse than NEWS. Best viewed in colour.

AP, average precision; AUROC, area under receiver operating characteristic curve; LightGBM, Light Gradient Boosted Machines; NEWS, National Early Warning Score.

**Figure 1:**
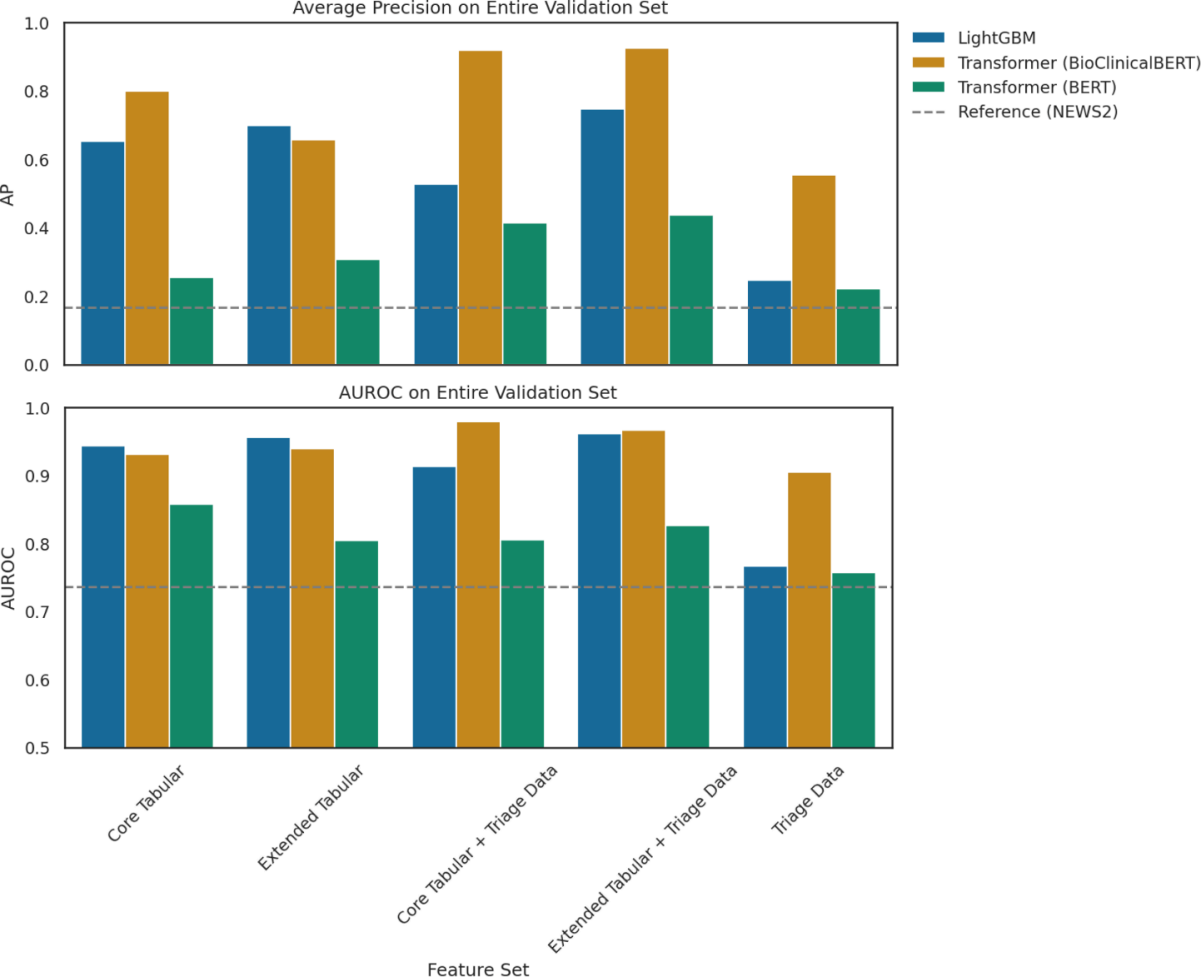
Average precision (top) and AUROC (bottom) of models predicting critical events with different sets of features. AP, average precision; AUROC, area under receiver operating characteristic curve. LightGBM, Light Gradient Boosted Machines. NEWS2, National Early Warning Score 2.

Figure 1 compares the AP and AUROC of all models. The relative stability of AUROC, juxtaposed with varying AP, is explained by the large class imbalance and motivated our focus on AP as the main evaluation metric. Figure 1 shows BioClincialBERT models generally outperformed tree-based models and increasing feature fidelity improved performance.

**Figure 2:**
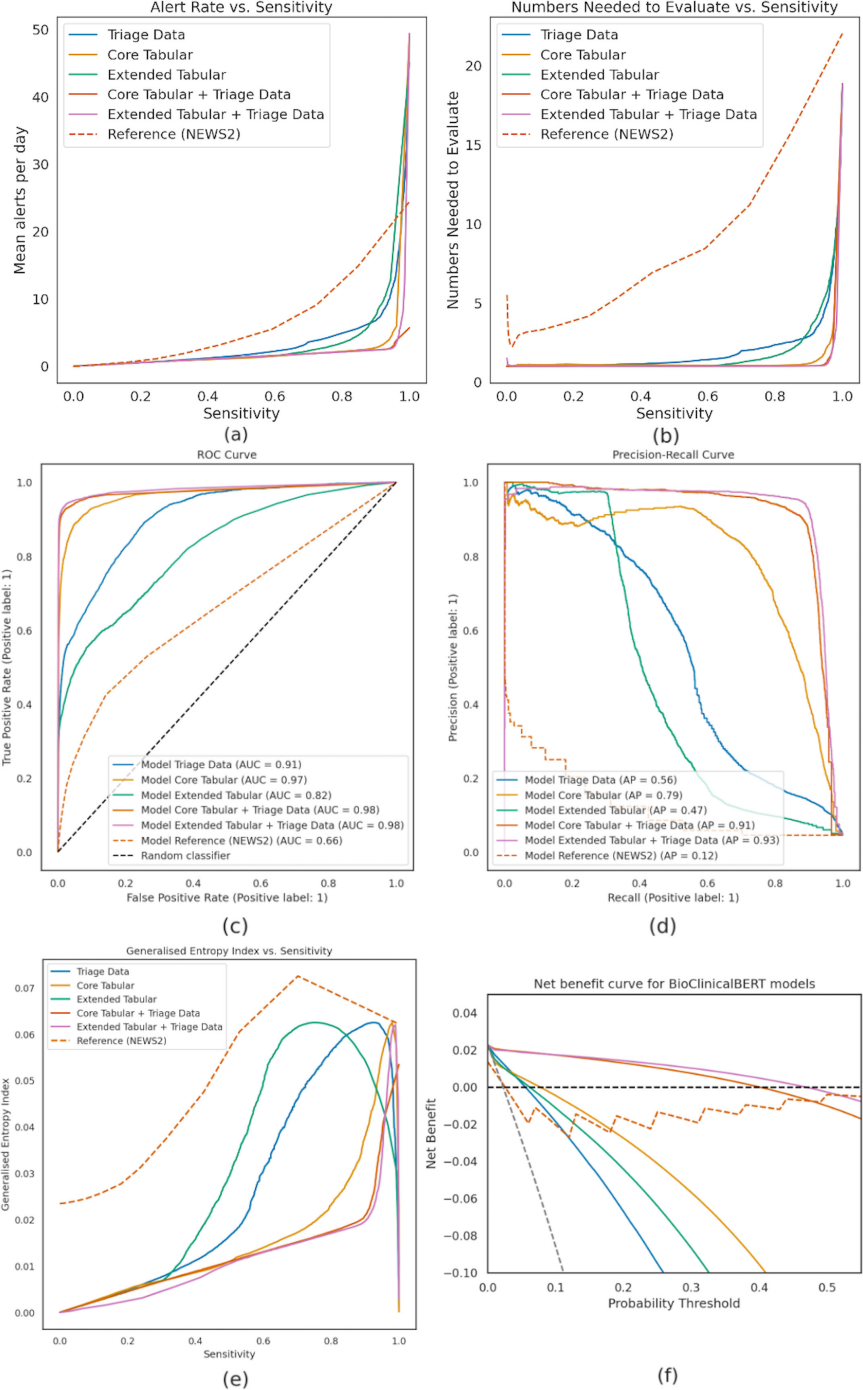
(**a**) Average number of daily alerts against model sensitivity. (**b**) Number needed to evaluate (right) against model sensitivity. (**c**) Receiver operating characteristic curves. (**d**) Precision-recall curves of BioClinicalBERT models. (**e**) Generalised entropy index (**I2**) versus sensitivity curves. (**f**) Decision (net benefit) curves for BioClinicalBERT models along with three reference strategies: treat all, treat none and NEWS. AP, average precision; AUC, area under curve; NEWS2, National Early Warning Score 2.AP, average precision; ROC, receiver operating characteristic.

**Figure 3:**
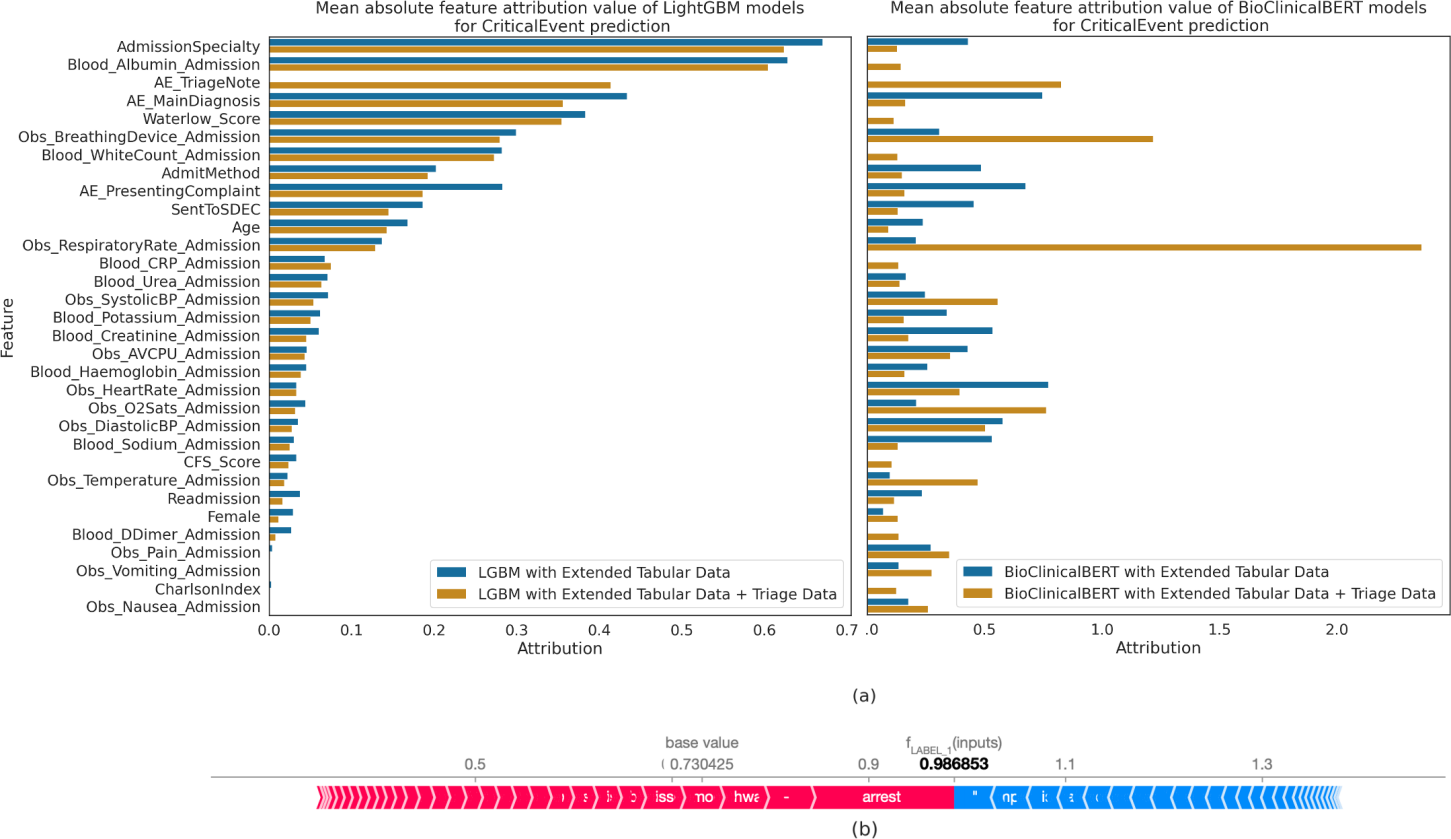
(**a**) Mean absolute feature importance of LGBM (left) and BioClinicalBERT (right) models, both with and without free-text fields. Note that, as the feature attribution method used is different for both LGBMs and BioClinicalBERT, direct comparisons between the two techniques are not necessarily valid. (**b**) Explainability values for a random sample from the validation set. This patient was correctly predicted by a finetuned BioClinicalBERT model as high risk for a critical deterioration. Words in red ‘push’ the model towards predicting critical deterioration and vice versa for blue words. The full-text input and its associated explainability have been redacted to preserve patient anonymity. CFS, Clinical Frailty Scale; CRP, C-reactive protein; LightGBM, Light Gradient Boosted Machines; AE, Accident and Emergency; Obs, observations.

Figure 2a,b shows the mean daily alerts and numbers needed to evaluate as model sensitivity increases. Figure 2c,d shows ROC and PR curves for BioClinicalBERT. Figure 3a compares feature importance of tree-based against transformer-based models, showing the mean absolute feature importance over the validation set. Note that direct explainability comparison between architectures may not be valid due to the different feature attribution methods used.

In contrast, figure 3b is a random local interpretability example from the validation set for BioClinicalBERT (more samples in onlinesupplemental figures 3^5^). Figure 3b only summarises local explainability; visualising individual word importance is possible but omitted here to preserve patient confidentiality.

Figure 2e presents the generalised entropy index *I*^2^ against sensitivity of BioClinicalBERT; lower *I*^2^ indicates lower levels of measured bias. Notably, all proposed models had lower *I*^2^ than NEWS, showing that our models produced less biased decisions.

Figure 2f reports the clinical benefit of our technique compared with NEWS; for any given decision threshold, the model with the highest net benefit has the theoretically highest clinical value.^24^ Figure 2f compares BioClinicalBERT against three baseline treatment strategies, treat all (everyone receives acute care treatment), treat none and NEWS (acute care treatment is delivered based on NEWS).

## Discussion

This study has demonstrated the effectiveness of modern deep learning for clinical decision support. Evaluating transformer-based techniques against classical methods showed free-text in EHR contains untapped predictive information that can augment decision support tools. Evaluating on temporal splits captured repeat attendees, reflecting clinical reality,^23^ though similar performance was demonstrated when removing repeat patients.

### Model performance analysis and comparison

All machine learning models vastly outperformed NEWS, with models using free-text triage data outperforming those without (BioClinicalBERT tabular only AP, 0.80; with free text:,0.92). The best model, BioClinicalBERT with extended tabular+triage notes, outperformed NEWS across all performance metrics (eg, sensitivity 0.92 vs 0.13 at specificity 0.99). Pretraining on relevant in-domain data was crucial; standard BERT greatly underperformed their medical terminology-orientated counterpart, BioClinicalBERT (AP: 0.31 vs 0.66 on the same features). We surmise that the standard BERT pretraining corpus does not reflect the specialist language used in this task, limiting its performance.

BioClinicalBERT’s substantial performance gains with text features supports triage notes containing underused information, likely capturing patients’ social context and diagnostic severity, which is difficult to represent in structured fields. Previous work supports the notion that clinical acumen, as captured in free-text comments, can help predict patient outcome. The Nurse Intuition Patient Deterioration Scale has greater AUROC than NEWS, while in combination with NEWS, it can enhance rapid response systems.^27^ Likewise, the Dutch-Early-Nurse-Worry-Indicator-Score suggests that ‘worried’ nurses can identify deteriorating patients before their physiological vital parameters start to deteriorate.^28^ While our triage notes may not explicitly discuss prognosis or worry, there is clinical evidence justifying their inclusion.

The BioClinicalBERT model using only triage notes and demographics performed comparably to models built on tabular features and outperformed NEWS. This suggests it may be possible to embed a model at admission using the earliest available data, allowing early risk stratification before awaiting other clinical data.

Although BioClinicalBERT showed significantly improved discriminative ability over LightGBMs (see online supplemental table 4 for final LightGBM parameters), the simpler LightGBM models require less computation and are more interpretable, so it may be more viable in clinical settings when performing similarly well to transformers. With only tabular data tree-based models matched transformer performance, suggesting trees may be preferred when lacking text data.

Alongside improved discriminative ability, our proposed methods demonstrably reduced alert rate compared with NEWS; BioClinicalBERT with triage notes reduced mean daily alerts (figure 2). Adding tabular data further reduced the alert rate and increased AUROC and AP, indicating fewer unnecessary alerts.

### Explainability and bias analysis

Using SHAP,^29^ we showed that complex models can be explained to clinicians, although with high computational cost. Figure 3 reveals that, without free-text triage notes, BioClinicalBERT relied more on primary admission diagnoses, presenting complaint and admission specialities, suggesting that this information is encapsulated within triage notes. Conversely, BioClinicalBERT incorporating triage notes placed greater importance on measured features (eg, vital signs); we hypothesise that this is because direct measurements cannot be inferred from triage notes. Interestingly, LightGBM models exhibited similar feature attributions regardless of free-text inclusion, suggesting limited free-text utilisation. In contrast to global explanations, we demonstrated how local explainability can provide patient-specific explanations to understand deterioration risk and guide patient management plan development.

Compared with NEWS, our models had lower *I*^2^ values across all sensitivity thresholds, indicating reduced bias. Generally, higher fidelity feature sets exhibited less bias than lower fidelity (figure 2e). However, this analysis is limited to our recorded protected characteristics. Future work should consider fine-grained data, such as socioeconomic and community context, which are known predictors of clinical risk,^30^ as language models can exhibit unfair bias.^31^

### Implications for deployment in a clinical context

As acute care data collection is not standardised, we made as few assumptions about the data as possible.^12^

Together with the methods’ handling of missing data, this supports our models’ generalisability across EHR. We demonstrated that machine learning risk prediction can be easily applied across different feature sets, showing they can be deployed to different hospitals despite varying data collection standards/procedures. Without the rigid data requirements of existing techniques, our methods are easier to deploy across settings.

We intentionally avoided setting classification thresholds, instead measuring discriminative skill; setting thresholds carries clinical, operational and ethical considerations.^25^ All of our models can be tuned to balance false-positive and false-negative outcomes based on healthcare provider/regulator preference. We see the adoption of machine learning models in clinical practice as *decision support tools* rather than *decision-making tools*. However, this must be appropriately balanced to combat alert fatigue.^30^ Our analysis showed that this is possible, as all models achieved fewer average daily alerts (figure 2a) and higher clinical utility or net benefit (figure 2f) than NEWS at all but the highest sensitivities. If deployed to match NEWS sensitivity, we would raise fewer alerts while achieving the same level of care. For example, fixing BioClinicalBERT with all feature sets to a sensitivity of 0.32 (matching NEWS ≥5) achieves a positive predictive value of 0.85 versus 0.18 for NEWS. Alternatively, if the decision threshold is softened to match the alert rate of NEWS, BioClinicalBERT would identify cases that NEWS would miss.

### Strengths and limitations

We believe this is the only large-scale evaluation of transformer-based models with free-text data as an EWS successor. We systematically examined how including free-text features improves model performance (increasing AP from 0.66 to 0.92), highlighting these untapped features’ usefulness. Importantly, we demonstrated that free-text notes alone contained sufficient predictive information to surpass existing EWS (AP ours, 0.92; AP NEWS, 0.12). Using explainability techniques, we demonstrated how explanations can elucidate important patient-level features, potentially increasing trust in the model and guiding clinical conversations.

Computing the generalised entropy index (*I*^2^), we compared the bias levels of our techniques against NEWS, showing our models yielded fairer distributions of benefit. However, data availability limited analysis to age, ethnicity and biological sex. Future research should consider other sources of bias such as socioeconomic status and free-text bias. Furthermore, our study contains data from a single site only. Data shift can affect the machine learning performance, and patient populations may vary significantly between hospitals;^13^ therefore, a multisite evaluation of our proposed techniques is warranted.

The use of free-text fields may differ between hospitals and requires further investigation; nomenclature, processes and data collection will differ between hospitals, possibly affecting model generalisability, necessitating a multicentre study. There were high rates of missing data, though reasons for this varied. Some were clinically meaningful, that is, the measurement was not clinically relevant. In other cases, values may not have been entered into the EHR correctly, perhaps because of operational pressures. We deliberately used models that can handle missing data, believing these yield techniques that are more applicable to real-world settings and allows for heterogeneity in features collected between hospitals. However, future studies should investigate the effect of missing data on the modelling process.

This study only showed the feasibility of using ML as an alternative to existing EWS. Prospective studies of our techniques are required to assess the impact of our models in clinical practice. These studies should consider factors such as usability and patient outcomes compared with existing EWS, together with patient and public involvement.

### Comparisons with other studies

Previous machine learning models have been proposed as EWS replacements,^32^ but to our knowledge, ours is the first to include free-text data. Our LightGBM models using only tabular features achieved higher performance than similar studies^16^ (AP our model, 0.75; AP previous, 0.53), while our best transformer-based techniques vastly outperformed them (AP ours, 0.92,;AP previous, 0.53). Recent systematic literature reviews^32^ report that many studies fail to report suitable metrics for imbalanced classification (eg, F1 or F2 score), instead reporting the AUROC metric which we demonstrated is unsuited to imbalanced data. Direct comparisons with previous studies are difficult due to obscured discriminative power, different test sets and varying critical event definitions.^32^ Notably, few prior studies have compared directly to existing EWS.^16 32^ Unlike previous studies,^32^ we have demonstrated explainability techniques and evaluated bias.

## Conclusion

Through experimentation on a large, real-world dataset, we demonstrated the feasibility of natural language modelling for clinical decision-support tasks and uncovered the untapped potential of unstructured free-text data in EHR. We evaluated our techniques’ bias, showing they are fairer than NEWS, and demonstrated how model explainability can augment clinical conversations. Such models are promising candidates to support decision-making and reduce critical event risk, greatly outperforming NEWS. We hope this encourages future researchers to include unstructured data in their modelling and supports deploying machine learning-based early warning systems in hospital.

## Supplementary material

10.1136/bmjhci-2024-101088 online supplemental file 1

10.1136/bmjhci-2024-101088 online supplemental figure 1

10.1136/bmjhci-2024-101088 online supplemental figure 2

10.1136/bmjhci-2024-101088 online supplemental figure 3

10.1136/bmjhci-2024-101088 online supplemental figure 4

10.1136/bmjhci-2024-101088 online supplemental figure 5

10.1136/bmjhci-2024-101088 online supplemental table 1

10.1136/bmjhci-2024-101088 online supplemental table 2

10.1136/bmjhci-2024-101088 online supplemental table 3

10.1136/bmjhci-2024-101088 online supplemental table 4

## Data Availability

Data are available upon reasonable request.
