# Supplementary material for: Performance of machine learning versus the national early warning score for predicting patient deterioration risk: a single-site study of emergency admissions
Source: BMJ Health Care Inform. 2024 Dec 4;31(1):e101088. doi: 10.1136/bmjhci-2024-101088 (PMC11624723; doi:10.1136/bmjhci-2024-101088)
Supplement: online supplemental file 1 [file bmjhci-31-1-s001.pdf]

1 Methods

This supplementary material expands on the details and explanations of the methods employed in this study. All methods are based upon established practice in machine learning, and so were omitted from the main paper; however, for reproducibility and the interested clinician, we include full explanations here.

1.1 Feature Sets

All features in the dataset were collected early during a patient's episode, although there are time variations for when they became available, for example, triage notes are typically available soon after arrival at hospital, while blood tests are not available until they have been analysed. We therefore evaluated models with incrementally richer data, including combinations of tabular and text features. We also experimented with using only freetext triage notes. This provided a better understanding of which features contain relevant information to each task and allowed for a better analysis of how, and where, the resulting models could be integrated into clinical practice. Table 1 lists the key feature combinations used. The *Core Tabular* feature set includes only those features present in a previous baseline study, [1] and was used for comparison of our models with a baseline. *Core Tabular* is designed to encapsulate features included in NEWS, with the addition of basic blood tests, demographic data, and diagnoses and admission routes. The *Extended Tabular* feature set contains all tabular features that were available in this study. All other features are grouped according to their type, i.e. text or tabular. In addition to these basic feature groups, combinations of feature groups are also used, for example, the combined feature group *Core Tabular* + *Triage Notes* includes the tabular features from *Core Tabular*, plus the freetext features from the *Triage Notes* group.

1.2 Data Augmentation and Feature Engineering

We engineered and/or augmented the following features:

- **ICD-10 codes**: we convert each originally recorded ICD-10 [2] three number code to its long-form English description, such as converting A00.1 to "Cholera due to *Vibrio cholerae* 01, biovar eltor".
- **Dates**: we removed dates as they can introduce bias to models. However, time-based features, such as length of stay, were kept.
- **30-day readmissions**: a Boolean value indicating records which are preceded by a record bearing the same unique patient ID and the two records' admission dates are $\leq$ 30 days apart.
- **Charlson Index**: a patient's Charlson Comorbidity Index Score, [3] calculated using comorbidities recorded in the coded diagnoses of the electronic health record (Supplementary Table 1)
- **Text embeddings**: we extracted text embeddings for the freetext triage notes columns using a pretrained BioClinicalBERT [4] model.

A lot of patient data is entered manually into the electronic health record, which can lead to transcription errors and potentially introduce bias into the models. Therefore, each vital sign was checked against fixed ranges, for example 0-100% for oxygen saturation, with acceptable thresholds determined by our clinical opinion (see Supplementary Table 2). Where data fell out of this range, we set it to the mid-point of the reported NEWS sub-score for the same record when known, for example, a record with a reported pulse of 460 and NEWS pulse sub-score of 1 would have their raw pulse value set to 45.

1.3 Machine Learning Pipeline

Both our tree-based and transformer-based models use the same training and evaluation pipeline:

1. Data pre-processing: the raw data is converted into vectors representing each clinical episode. The exact features included are combinations of the feature sets listed in Table 1. *Optional:* When using transformer-based models, each episode vector is converted to text, in the format `Column name1: Value1, Column name2: Value2...Column nameN: ValueN`. For example: `Waterlow Score: 2, Blood Creatinine Admission: 100...`
2. Data splitting: the pre-processed data is partitioned into two subsets, one for model training and the other for validation. We opted for a temporal train-test split over standard random splitting [5] and partitioned the dataset such that the first 2/3 of records chronologically serve as the training set and the latter 1/3 as the validation set. For some experiments we excluded any validation set records where the patient, as identified by their unique ID, had also appeared in the training set in a previous admission.

3. Model training: the chosen model architecture is trained. The architectures and their training algorithms are outlined in Supplementary Sections 1.3.1 and 1.3.2.

4. Model calibration: *optional*. Tree-based models benefit from a post-processing step called calibration. This maps the numerical outputs of the trained model into well-calibrated probabilities such that the model's output $F(x)$ becomes an estimate of $P(y=1|x)$. For this, we used isotonic calibration. [6]

5. Model evaluation: the trained model is evaluated on the unseen validation dataset specified in the second step of the model pipeline, using a set of standard metrics. These metrics, and the reasoning for their inclusion, are explained in Section 2.5 and Supplementary Section 1.4.

1.3.1 Tree-Based Architectures and Training

Hyperparameter optimisation for the LightGBM models used a Bayesian optimisation process that sweeps over the space of possible hyperparameters and selects values which maximise average precision (AP). [7] The final model was constructed using the best hyperparameters after 1000 iterations (see Supplementary Table 4 for full details of sweep parameters and hyperparameters used).

Model calibration was combined with k-fold cross-validation, randomly splitting the training dataset into $k=5$ equal-sized partitions. Each subset was used as a validation set (for model calibration) exactly once; we trained a LightGBM model on four subsets and use the remaining subset to fit the calibrator. This resulted in 5 independent models that form sub-models of a final, calibrated ensemble of LightGBM models, such that the final predicted probability of the ensemble, C, is the arithmetic mean of the sub-models' output. As discussed in Section 2.3 and Section 1.3, when training LightGBMs on text data, we first converted the freetext *Triage Note* and *Presenting Complaints* features to tabular features using text embeddings generated from a BioClinicalBERT model. [4] This results in a 768-vector that represents the text for a given dataset record. This vector was then appended to the structured data already extracted from the electronic health record.

1.3.2 Clinical Language Models and Training

Text-based transformer models are typically pre-trained on a large corpus of text data. While this training text is usually general-purpose, [8] it can also be task specific, [9] for example, healthcare-related text corpora, because models pre-trained on task-specific data will learn higher quality associations about the specific task at hand, thus performing better. To assess the extent to which this domain-specific training affects our models, we experimented with two differently pretrained versions of BERT [8]. Specifically, we considered:

- **BERT**: [8] The original BERT model, pretrained on a large corpus of English text
- **BioClinicalBERT**: [4] An instance of BioBERT [10] which has been further trained on clinical notes contained within the MIMIC-III [11] dataset

These models contain a spread of knowledge, with one being more general while the other is equipped with specific clinical information. We tokenised all textual features, converting their text into token sequences. For this we used WordPiece [8], a model-based subword segmentation algorithm pre-trained on English corpora to recognise around 30,000 common sub-word tokens. All transformer models were then finetuned on our training dataset for a total of 10 epochs, with a learning rate of 2×10^{-5} and weight decay of 0.01; the best model checkpoint was then chosen to maximise AP. Following current standard practice, we did not calibrate the output of BERT-based models. [12]

1.4 Evaluation Metrics

Average precision (AP) is calculated by plotting the precision-recall curve (PR curve) and calculating the area under the curve. Precision is the ratio $\frac{TP}{TP+FP}$ and recall is $\frac{TP}{TP+FN}$ where TP is the number of true positives, FP the number of false positives and FN the number of false negatives. An unskilled model would present a horizontal line at $y=\frac{P}{P+N}$, where P,N are the number of positive and negative, respectively, samples in the dataset. A theoretical perfect model would yield a single point at $(1,1)$. The PR curve further allowed the visual inspection of how quickly positive predictive value (PPV) deteriorated as model sensitivity was increased, which is helpful in a task where it may be appropriate to value sensitivity over specificity.

The strong class imbalance in our task makes the PR curve a much better indicator of performance than the traditional receiver operating characteristic (ROC) curve, [13] as the latter does not consider the ability of a classifier to accurately classify the minority class(es). [14] However, we still included ROC curves and area under the ROC (AUROC) metrics in our analyses to allow comparisons with other studies. To construct a ROC curve, the false positive rate (FPR) is plotted on the x-axis against the sensitivity on the y-axis. A completely random classifier will plot the line $y=x$, with a corresponding AUROC of 0.5, with an AUROC of ≥ 0.8 indicating a good classifier. [15] We also report the specificity of the model, which is the sensitivity of the negative class.

To give an indication of possible model performance when a threshold is set and acknowledging standard reporting practices, we also compute F2 scores under a decision threshold of 0.5, i.e., samples with a predicted probability ≥ 0.5 are predicted as high risk. F2 is the harmonic mean of precision and recall, with recall weighted by a factor 2. We opted for the F2 score over the commonly used F1 metric as, when predicting patients at risk of critical deterioration, reducing the number of false negatives is more important than reducing the number of false positives.

We additionally assessed the clinical net benefit, [16] which is calculated by plotting the decision curve produced by a model, threshold versus net benefit. Net benefit is defined as:

$$\text{net benefit} = \text{sensitivity} \times \text{prevalence} - (1 - \text{specificity}) \times (1 - \text{prevalence}) \times \text{odds}$$

where 'odds' is the odds value at a given threshold probability, and sensitivity and specificity are also calculated at the same threshold probability. Hence, the unit of net benefit is true positives; for example, a net benefit of 0.07 is 7 true positives per 100 interventions. Net benefit differs from other metrics as it incorporates the consequences of decisions that may be made because of the model. The most basic interpretation of the decision curve produced by a net-benefit analysis is that the model with the highest net benefit at a particular threshold has the highest clinical value. In our analysis we compared four scenarios, selecting all patients for the intervention (treat all patients as high-risk), selecting no patients (treat none, i.e. no patients considered high-risk), selecting patients based on NEWS, and selecting patients using our predictive model. Finally, we compared the daily alert rate and numbers needed to evaluate (NNE) to detect one deteriorating/high-risk patient of our models to those of NEWS across a range of sensitivities.

1.4.1 Model Explainability

For LightGBM-based models, we used the TreeSHAP approximation to calculate SHAP values, whereas for transformer-based models we used GradientSHAP. [17] Both computational and methodological limitations inhibit our ability to calculate global feature importance values for transformer architectures. [18, 19] Therefore, for models primarily based on freetext, we focused on generating local, patient-individual explanations instead, using ParitionSHAP. [17] For transformer-based models that utilise tabular features, we then produced a global view of their learned associations by averaging the absolute feature attribution values of each sample in the validation set. As we used a different feature attribution calculation method for each model architecture, it is not possible to directly compare attribution values between the two techniques.

1.4.2 Model Bias

Given a patient record x_i with ground-truth y_i, the benefit experienced by the patient due to model prediction $M(x_i)$ is defined as

$$b_i = M(x_i) - y_i + 1$$

Under this representation, a false-positive patient experiences a large benefit ($b = 2$), while a false-negative patient is given the largest penalty ($b = 0$). Given a vector of benefit values $b = (b_1, b_2, ..., b_n)$ and their arithmetic mean μ, we then define the generalised entropy index I^α as:

$$I^\alpha(b) = \frac{1}{n\alpha(\alpha-1)} \sum_{i=1}^{n} \left(\frac{b_i}{\mu}\right)^\alpha - 1$$

For I^α, the ideal value is 0, representing completely fair classifier, while higher values indicate worsening levels of bias.

References

1. S. Boulitsakis Logothetis, D. Green, M. Holland, and N. Al Moubayed, "Predicting Acute Clinical Deterioration with Interpretable Machine Learning to support Emergency Care Decision Making," preprint, Scientific Reports (Preprint), Dec. 2022.
2. World Health Organization, "Icd-10 : international statistical classification of diseases and related health problems : tenth revision," 2004.
3. H. Quan, B. Li, C. M. Couris, K. Fushimi, P. Graham, P. Hider, J.-M. Januel, and V. Sundararajan, "Updating and validating the charlson comorbidity index and score for risk adjustment in hospital discharge abstracts using data from 6 countries," *Am. J. Epidemiol.*, vol. 173, pp. 676–682, Mar. 2011.
4. E. Alsentzer, J. R. Murphy, W. Boag, W.-H. Weng, D. Jin, T. Naumann, and M. McDermott, "Publicly available clinical bert embeddings," *arXiv preprint arXiv:1904.03323*, 2019.
5. D. G. Altman and P. Royston, "What do we mean by validating a prognostic model?," *Statistics in medicine*, vol. 19, no. 4, pp. 453–473, 2000.

6. A. Niculescu-Mizil and R. Caruana, “Predicting good probabilities with supervised learning,” in *Proceedings of the 22nd international conference on Machine learning*, pp. 625–632, 2005.
7. J. Bergstra, R. Bardenet, Y. Bengio, and B. Kégl, “Algorithms for hyper-parameter optimization,” *Advances in neural information processing systems*, vol. 24, 2011.
8. J. Devlin, M.-W. Chang, K. Lee, and K. Toutanova, “Bert: Pre-training of deep bidirectional transformers for language understanding,” *arXiv preprint arXiv:1810.04805*, 2018.
9. K. S. Kalyan, A. Rajasekharan, and S. Sangeetha, “Ammus: A survey of transformer-based pretrained models in natural language processing,” *arXiv preprint arXiv:2108.05542*, 2021.
10. J. Lee, W. Yoon, S. Kim, D. Kim, S. Kim, C. H. So, and J. Kang, “Biobert: a pre-trained biomedical language representation model for biomedical text mining,” *Bioinformatics*, vol. 36, no. 4, pp. 1234–1240, 2020.
11. A. E. Johnson, T. J. Pollard, L. Shen, L.-w. H. Lehman, M. Feng, M. Ghassemi, B. Moody, P. Szolovits, L. Anthony Celi, and R. G. Mark, “Mimic-iii, a freely accessible critical care database,” *Scientific data*, vol. 3, no. 1, pp. 1–9, 2016.
12. S. Desai and G. Durrett, “Calibration of pre-trained transformers,” *arXiv preprint arXiv:2003.07892*, 2020.
13. N. A. Obuchowski and J. A. Bullen, “Receiver operating characteristic (roc) curves: review of methods with applications in diagnostic medicine,” *Physics in Medicine & Biology*, vol. 63, no. 7, p. 07TR01, 2018.
14. T. Saito and M. Rehmsmeier, “The precision-recall plot is more informative than the roc plot when evaluating binary classifiers on imbalanced datasets,” *PloS one*, vol. 10, no. 3, p. e0118432, 2015.
15. G. B. Smith, D. R. Prytherch, P. Meredith, P. E. Schmidt, and P. I. Featherstone, “The ability of the national early warning score (news) to discriminate patients at risk of early cardiac arrest, unanticipated intensive care unit admission, and death,” *Resuscitation*, vol. 84, no. 4, pp. 465–470, 2013.
16. A. J. Vickers, B. van Calster, and E. W. Steyerberg, “A simple, step-by-step guide to interpreting decision curve analysis,” *Diagnostic and prognostic research*, vol. 3, no. 1, pp. 1–8, 2019.
17. S. M. Lundberg and S.-I. Lee, “A unified approach to interpreting model predictions,” *Advances in neural information processing systems*, vol. 30, 2017.
18. H. Chefer, S. Gur, and L. Wolf, “Transformer interpretability beyond attention visualization,” *CoRR*, vol. abs/2012.09838, 2020.
19. S. Abnar and W. H. Zuidema, “Quantifying attention flow in transformers,” *CoRR*, vol. abs/2005.00928, 2020.
