# Supplementary figures and images for: Performance of machine learning versus the national early warning score for predicting patient deterioration risk: a single-site study of emergency admissions
Source: BMJ Health Care Inform. 2024 Dec 4;31(1):e101088. doi: 10.1136/bmjhci-2024-101088 (PMC11624723; doi:10.1136/bmjhci-2024-101088)

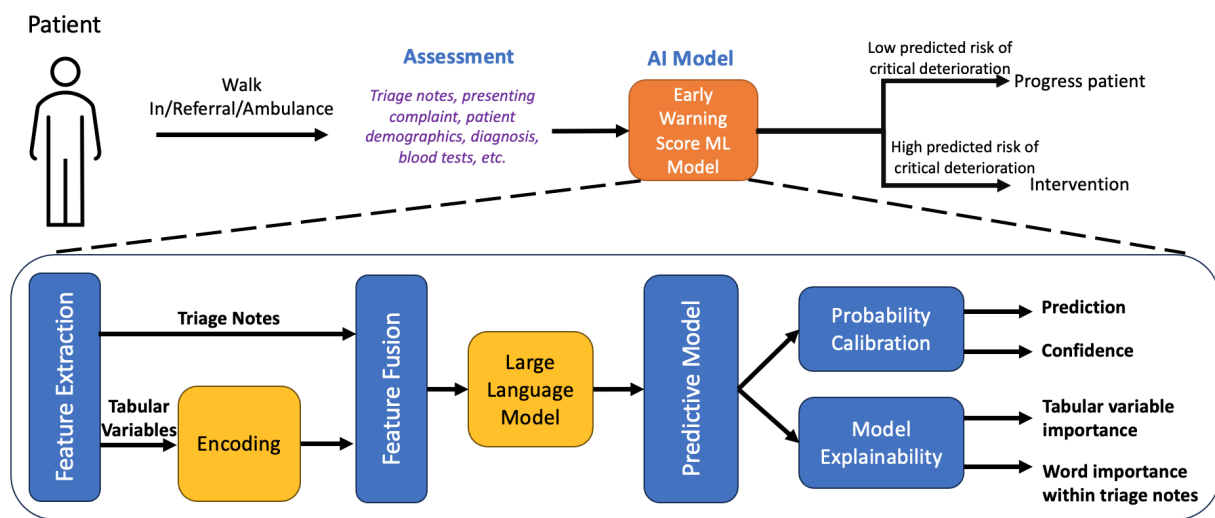

**Figure 1.** Inference flowchart of our final, best performing model. Best viewed in colour.

Supplement: online supplemental figure 1 [file bmjhci-31-1-s002.pdf]

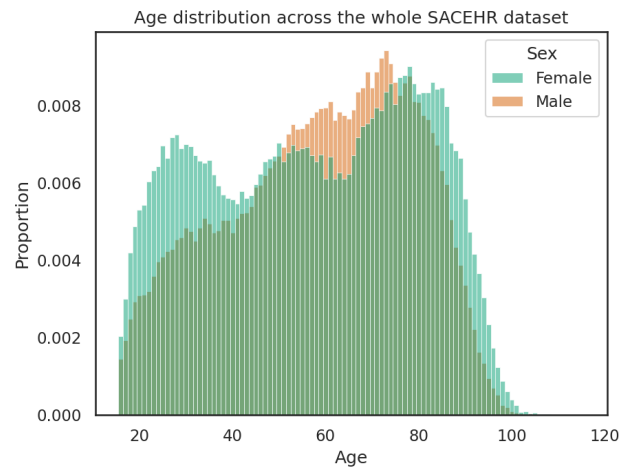

**Figure 2.** Distribution of patient age and sex across the entire dataset.

Supplement: online supplemental figure 2 [file bmjhci-31-1-s003.pdf]
