# Supplementary material for: Performance of machine learning versus the national early warning score for predicting patient deterioration risk: a single-site study of emergency admissions
Source: BMJ Health Care Inform. 2024 Dec 4;31(1):e101088. doi: 10.1136/bmjhci-2024-101088 (PMC11624723; doi:10.1136/bmjhci-2024-101088)
Supplement: online supplemental table 1 [file bmjhci-31-1-s007.pdf]

**Table 1.** Features collected by the electronic health record system

| Feature | Description |
|---|---|
| Demographics and metadata | age, gender, ethnicity, admission/discharge dates, discharge destination, 30-day mortality |
| Unstructured freetext fields | triage notes, presenting complaints |
| Clinical scales | AVCPU score (Awake, Verbal, Confusion, Pain, Unresponsive; a measure of level of consciousness included in NEWS), Waterlow Score, Clinical Frailty Score |
| Admission pathway | admission route (e.g., ambulance, emergency department self-attender, emergency GP referral) and their admitting specialty (e.g., acute internal medicine, emergency medicine). |
| Vital signs and their associated NEWS scores | body temperature (°C), heart rate (beats/min), systolic and diastolic blood pressure (mmHg), and peripheral oxygen saturation (%). These data points are first recorded within a target of 30 minutes from arrival at the hospital, and then periodically throughout a patient's stay. |
| Blood tests | haemoglobin (mmol/L), urea (mmol/L), sodium (mmol/L), potassium (mmol/L), creatinine ($\mu$mol/L), D-dimer (ng/mL FEU), CRP (mg/L), albumin (g/L), white blood cells (cells $\times 10^9$/L) |
| Diagnoses | main ICD10 diagnosis, and up to 15 secondary ICD10 diagnoses |
| Procedures | main OPCS-4 procedure and up to 15 secondary procedures |
| Ward utilisation | each ward that the patient was sequentially admitted to during their in-patient episode |
