# Supplementary material for: Performance of machine learning versus the national early warning score for predicting patient deterioration risk: a single-site study of emergency admissions
Source: BMJ Health Care Inform. 2024 Dec 4;31(1):e101088. doi: 10.1136/bmjhci-2024-101088 (PMC11624723; doi:10.1136/bmjhci-2024-101088)
Supplement: online supplemental table 2 [file bmjhci-31-1-s008.pdf]

Table 2. Valid ranges for manually recorded data features.

Variable	Range	Unit
SpO2	40-100	%
Systolic BP	40-300	mmHg
Diastolic BP	20-200	mmHg
Temperature	25-45	°C
Pulse	35-300	Beats/min
Respiration Rate	5-80	Breaths/min
