# Supplementary material for: Performance of machine learning versus the national early warning score for predicting patient deterioration risk: a single-site study of emergency admissions
Source: BMJ Health Care Inform. 2024 Dec 4;31(1):e101088. doi: 10.1136/bmjhci-2024-101088 (PMC11624723; doi:10.1136/bmjhci-2024-101088)
Supplement: online supplemental table 3 [file bmjhci-31-1-s009.pdf]

Table 3. Full table of results for all LightGBM, BERT and BioClinicalBERT models tested on the validation set with repeat attendees included in the training set removed. AUROC: Area Under Receiver Operating Characteristic Curve; AP: Average Precision

Model Architecture	Features	Precision	Recall	AUROC	F2	Specificity	AP
LightGBM	Core Tabular	0.9361	0.7618	0.9742	0.7913	0.9961	0.8868
	Extended Tabular	0.9345	0.7638	0.9774	0.7928	0.9960	0.8917
	Core Tabular + Text Embeddings	0.9527	0.7490	0.9746	0.7824	0.9972	0.8895
	Extended Tabular + Text Embeddings	0.9456	0.7490	0.9770	0.7815	0.9967	0.8937
	Text Embeddings	0.7967	0.1203	0.8603	0.1449	0.9976	0.4008
BioClinicalBERT	Core Tabular	0.9035	0.9141	0.9850	0.9121	0.9939	0.99262
	Extended Tabular	0.7191	0.8804	0.9812	0.8426	0.9739	0.90942
	Triage Notes + Demographics	0.3723	0.9039	0.9472	0.7031	0.8847	0.6506
	Core Tabular + Triage Notes	0.9960	0.9868	0.9964	0.9886	0.9997	0.9926
	Extended Tabular + Triage Notes	0.9679	0.9564	0.9902	0.9584	0.9976	0.9691
BERT	Core Tabular	0.2203	0.8761	0.8630	0.7706	0.9389	0.2697
	Extended Tabular	0.1268	0.9297	0.7774	0.4104	0.5164	0.1424
	Triage Notes + Demographics	0.2524	0.5415	0.7579	0.4890	0.9247	0.2161
	Core Tabular + Triage Notes	0.4796	0.8717	0.8597	0.7492	0.9285	0.4511
	Extended Tabular + Triage Notes	0.5551	0.8687	0.8683	0.7805	0.9473	0.4720
