# Supplementary material for: Performance of machine learning versus the national early warning score for predicting patient deterioration risk: a single-site study of emergency admissions
Source: BMJ Health Care Inform. 2024 Dec 4;31(1):e101088. doi: 10.1136/bmjhci-2024-101088 (PMC11624723; doi:10.1136/bmjhci-2024-101088)
Supplement: online supplemental table 4 [file bmjhci-31-1-s010.pdf]

Table 4. Hyperparameters used for the LightGBM models, chosen after 1000 iterations of Bayesian optimisation.

Parameter	Core Tabular	Extended Tabular	Core Tabular + Triage Notes	Extended Tabular + Triage Notes	Triage Notes	Sweep Range
colsample_bytree	0.5039	0.7830	0.9384	0.9109	0.4202	$[0.01, 1]$
is_unbalance	True	True	True	True	True	$\{\text{True}, \text{False}\}$
min_child_samples	100	90	83	104	154	$[5, 1000]$
num_leaves	13	20	29	13	32	$[2, 50]$
reg_alpha	0.3942	0.5835	1.840	0.5834	0.0583	$[0, 100]$
reg_lambda	0.9894	0.0049	0.5930	0.0048	0.0384	$[0, 100]$
subsample	0.7304	0.7583	0.4827	0.5839	0.9834	$(0, 1]$
subsample_freq	4	4	4	4	4	$[0, 10]$
