# Supplementary material for: Performance of machine learning versus the national early warning score for predicting patient deterioration risk: a single-site study of emergency admissions
Source: BMJ Health Care Inform. 2024 Dec 4;31(1):e101088. doi: 10.1136/bmjhci-2024-101088 (PMC11624723; doi:10.1136/bmjhci-2024-101088)
Supplement: online supplemental figure 3 [file bmjhci-31-1-s004.pdf]

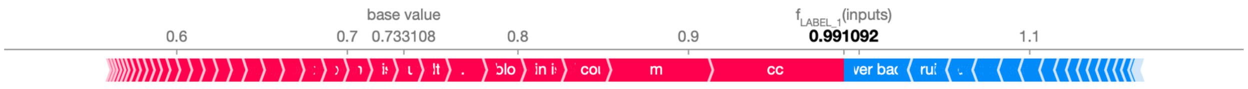

Figure 3. Explainability values for a random sample from the validation set. This patient was correctly predicted by a finetuned BioClinicalBERT model as high risk for a critical deterioration. Words in red ‘push’ the model towards predicting critical deterioration, and vice versa for blue words. The full text input and its associated explainability have been redacted for patient anonymity.
